# The downregulated drug-metabolism related ALDH6A1 serves as predictor for prognosis and therapeutic immune response in gastric cancer

**DOI:** 10.18632/aging.204270

**Published:** 2022-09-12

**Authors:** Yuan Cai, Rong Zeng, Jinwu Peng, Wei Liu, Qingchun He, Zhijie Xu, Ning Bai

**Affiliations:** 1 Department of Pathology, Xiangya Hospital, Central South University, Changsha 410008, Hunan, China; 2 General Surgery Department, Second Xiangya Hospital, Central South University, Changsha 410008, Hunan, China; 3 Department of Pathology, Xiangya Changde Hospital, Changde 415000, Hunan, China; 4 National Clinical Research Center for Geriatric Disorders, Xiangya Hospital, Central South University, Changsha 410008, Hunan, China; 5 Department of Orthopedic Surgery, The Second Hospital University of South China, Hengyang 421001, Hunan, China; 6 Department of Emergency, Xiangya Hospital, Central South University, Changsha 410008, Hunan, China; 7 Department of Emergency, Xiangya Changde Hospital, Changde 415000, Hunan, China; 8 Department of General Surgery, Xiangya Hospital, Central South University, Changsha 410008, Hunan, China

**Keywords:** ALDH6A1, gastric cancer, drug-metabolism, prognosis, immune regulation

## Abstract

Drug metabolism-associated genes have been clarified to play a vital role in the process of cancer cell growth and migration. Nevertheless, the correlation between drug metabolism-associated genes and gastric cancer (GC) has not been fully explored and clarified. This paper has focused on the role of aldehyde dehydrogenase 6 family member A1 (ALDH6A1), a drug metabolism-associated gene, in the immune regulation and prognosis of GC patients. Using several bioinformatics platforms and immunohistochemistry (IHC) assay, we found that ALDH6A1 expression was significantly down-regulated in GC tissues. Moreover, higher expression of ALDH6A1 was related to the better prognosis of GC patients. ALDH6A1 was also found to be involved in the regulation of several immune-associated signatures, including immunoinhibitors. In conclusion, the above results have concluded that aberrant expression of ALDH6A1 might be served as the promising predictor for prognosis and clinical immunotherapy response in GC patients.

## INTRODUCTION

Gastric cancer (GC) is a common death cause worldwide. Although the incidence rate of gastric cancer has decreased recently, the mortality rate is still ranked as the third place all over the world [1, 2]. The H. pylori infection has been reported to be the main reason for gastric cancer patients, accounting for about 89% [3]. Nowadays, there are several approaches that are applied for the treatment of gastric cancer patients, including surgical treatment, chemotherapy, radiotherapy and immunotherapy [4]. However, the prognosis of GC patients who are diagnosed at an advanced disease stage is poor. Consequently, it is significantly crucial to explore a novel molecular to improve the prognosis of GC patients.

Recently, studies have shown that immune cells of tumor microenvironment (TME) could play an essential role in the process of inhibiting or inducing tumors. Furthermore, the molecules of TME could trigger the immune response and promote tumor progression [5, 6]. Moreover, a better understanding of TME could improve the predictive prognostic value of GC patients [7]. It is significantly important to further explore the underlying mechanisms of immune systems and the relationship between TME and gastric cancer patients.

Drug metabolism has been reported to be a procedure that drugs are metabolized and used via enzymes. The bioavailability of drugs could be affected by the activity of drug metabolism enzymes [8]. The investigation of drug metabolism will be beneficial for the drug employment and clinical therapy of human diseases [9]. Additionally, the interventions of metabolism could induce immune response and benefit the immunotherapy concerning various kinds of cancers [10]. Aldehyde dehydrogenase 6 family, member A1 (ALDH6A1) is verified to participate in the metabolism of leucine, valine and isoleucine. Moreover, ALDH6A1 has been reported to be involved in the process of cancers. ALDH6A1, as one of a differentially expressed genes (DEGs) of kidney renal clear cell carcinoma (ccRCC) statistics obtained from the TCGA database. And the overexpression level of ALDH6A1 could inhibit cell growth and migration of renal cancer cells. Another study has demonstrated that the inhibition of ALDH6A1 might have a strong link with unusual liver cancer cell proliferation [11, 12]. Whereas, there is no study that focuses on the relationship between gastric cancer patients and the expression level of ALDH6A1.

Our paper will investigate the roles of downregulated ALDH6A1 in GC by several bioinformatic platforms. Higher expression level of ALDH6A1 could be strongly related to better prognosis of GC patients. Meanwhile, ALDH6A1 expression was verified to be linked with the immune infiltration of lymphocytes. The above results have demonstrated that ALDH6A1 could be a new prognostic biomarker for GC patients.

## MATERIALS AND METHODS

### Data collection

We applied Gene Expression Omnibus (GEO) platform [13] to explore two datasets concerning gastric cancer. And we obtained the statistics of GSE26942 [14] and GSE33651 [15] from the database (Table 1). Additionally, we have explored the differentially expressed genes (DEGs) of the normal group and gastric cancer group. We set up p < 0.05 as statistically significant. And the |logFC| was set as follows: |logFC| ≥ 1.0. To further investigate the co-differently expressed genes among the two datasets and the drug metabolism-associated gene dataset, we have employed the Venn plot.

**Table 1:** The features of the two GEO datasets about gene expression profiling by array.

**GEO^a^ datasets**	**Platform**	**Sample size**		**DEGs^b^**	**References**
		**cancer**	**normal**		
GSE26942	GPL6947	202	12	60 up-regulated genes and 357 down-regulated genes	[14]
GSE33651	GPL2895	40	12	630 up-regulated genes and 73 down-regulated genes	[15]

^a^GEO, Gene Expression Omnibus datasets.

^b^DEGs, differentially expressed genes.

### Bioinformatics platforms

In order to analyze the expression and immune response of ALDH6A1 in gastric cancer patients, several platforms were used (Table 2). In the first place, the Kaplan-Meier plotter [16] was applied to analyze the prognostic values of ALDH6A1 in GC. The platform was employed to investigate the overall survival (OS), post progression survival (PPS) and first-progression (FP) of the co-DEGs in gastric cancer patients. In addition to this, the TCGA database, the GEPIA2.0 platform [17] and the TNMplot [18] were used to compare the ALDH6A1 expression between the normal group and tumor group. Furthermore, the correlation between the expression level of ALDH6A1 and clinical characteristic parameters of GC patients have been explored and illustrated (Table 3). Then, the co-expressed network of ALDH6A1 was evaluated via LinkedOmics database [19]. We have investigated the Gene Ontology (GO) signaling pathway and Kyoto Encyclopedia of Genes and Genomes (KEGG) signaling pathway through the LinkedOmics platform. After then, the TISIDB platform [20] was employed to explore the relationship between ALDH6A1 expression and immune regulation. And last, the link between ALDH6A1 and VSIR have been clarified.

**Table 2:** Bioinformatics platforms that are used to evaluate the significance of ALDH6A1 in gastric cancer.

**Database**	**URL**	**Refs**
GEO	https://www.ncbi.nlm.nih.gov/gds/?term=	[13]
Kaplan-Meier Plotter	http://kmplot.com/analysis/	[16]
TNMplot	http://www.tnmplot.com	[18]
GEPIA2.0	http://gepia.cancer-pku.cn/	[17]
LinkedOmics	http://www.linkedomics.org/admin.php	[19]
TISIDB	http://cis.hku.hk/TISIDB/	[20]

**Table 3:** The relationship between the expression of ALDH6A1 and clinical characteristic parameters in GC patients from TCGA.

**Characteristics**	**Total (N)**	**Odds ratio (OR)**	**P value**
T stage (T3&T4&T2 vs. T1)	367	0.341 (0.108-0.913)	0.043
N stage (N1&N2&N3 vs. N0)	357	1.191 (0.761-1.870)	0.445
M stage (M1 vs. M0)	355	0.643 (0.272-1.457)	0.296
Pathologic stage (Stage III&Stage IV&Stage II vs. Stage I)	352	0.762 (0.419-1.368)	0.364
Gender (Male vs. Female)	375	0.919 (0.602-1.402)	0.695
Age (>65 vs. <=65)	371	0.907 (0.602-1.367)	0.642
H pylori infection (Yes vs. No)	163	0.650 (0.236-1.741)	0.392
Barretts esophagus (Yes vs. No)	208	0.814 (0.275-2.353)	0.701
Reflux history (Yes vs. No)	214	0.739 (0.365-1.481)	0.394
Histologic grade (G3&G2 vs. G1)	366	1.023 (0.280-3.736)	0.972
Residual tumor (R2&R1 vs. R0)	329	1.135 (0.541-2.420)	0.738
Antireflux treatment (Yes vs. No)	179	0.445 (0.208-0.927)	0.033

### Immunohistochemistry (IHC) assay

We collected GC tissues and corresponding normal tissues from Department of Pathology, Xiangya Hospital, Central South University. Then, we conducted IHC analysis to investigate the expression levels of ALDH6A1 in the tumor tissues and normal tissues.

### Statistical evaluation

In this paper, the results were described as mean ± standard deviation (SD). And t-test was applied to compare the difference of the normal group and tumor group. We considered P < 0.05 as statistically significant.

## RESULTS

### The differentially expressed genes between gastric cancer and normal tissues

**Figure 1:**
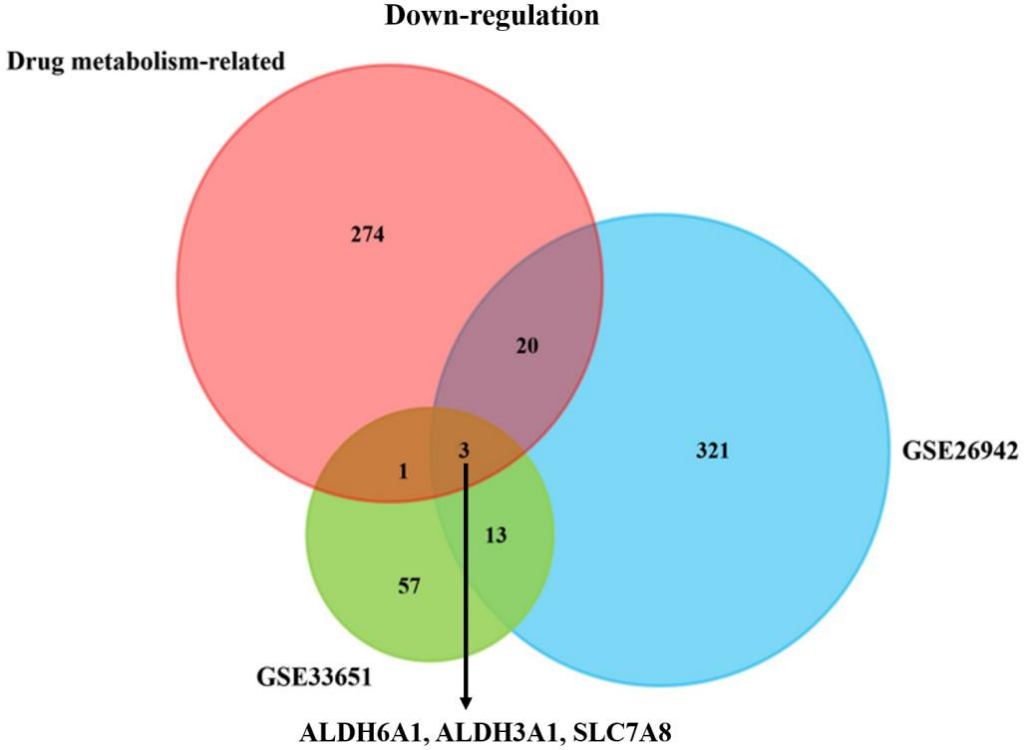
**The downgulated co-DEGs in the drug-metabolism related geneset and two GC datasets.** The Venn plot depicting three downregulated drug-metabolism related genes (ALDH6A1, ALDH3A1 and SLC7A8) could be correlated with the GC patients’ progression.

We explored two GC datasets from the GEO database and downloaded the statistics of the two datasets. The p-value < 0.05 was regarded as statistically significant. And the |logFC| was set as: |logFC| ≥ 1.0. The datasets were both divided into two groups: the normal group and gastric cancer group. Then, we could find that there existed 60 up-regulated genes and 357 down-regulated genes in the GSE26942 dataset. Moreover, there existed 630 up-regulated genes and 73 down-regulated genes in the GSE33651 dataset (Supplementary Table 1). Furthermore, the Venn plot (http://bioinformatics.psb.ugent.be/webtools/Venn/) was employed to explore the importance of the drug metabolism-associated genes in gastric cancer. As Figure 1 portrayed, three co-down-regulated genes, including ALDH6A1, ALDH3A1 and SLC7A8, could be important in the development of GC. And we found no co-up-regulated genes between the three datasets (Supplementary Figure 1).

### Kaplan-Meier plotter depicting the prognostic values of ALDH6A1 in gastric cancer

**Figure 2:**
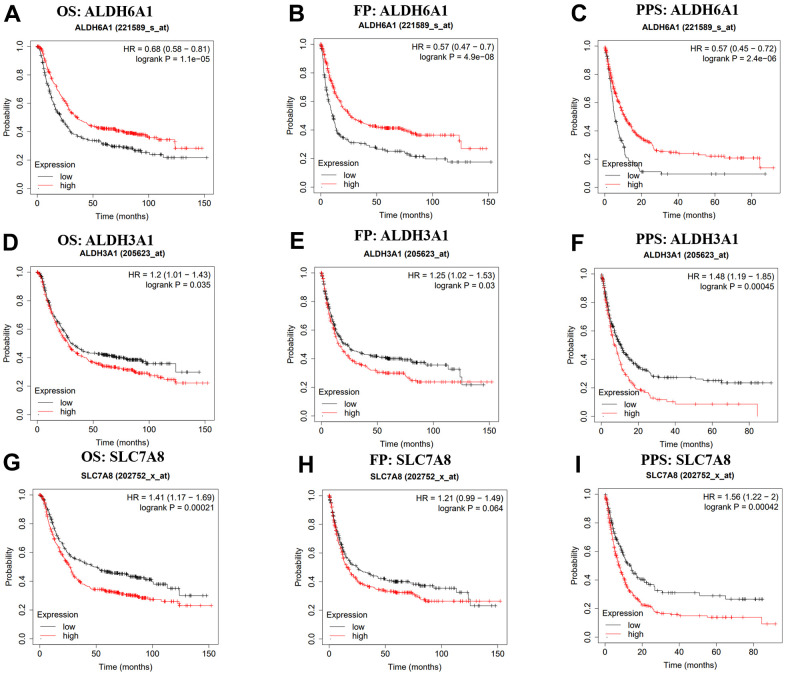
**Kaplan-Meier platform showing the prognostic values of ALDH6A1, ALDH3A1 and SLC7A8 in GC.** (**A**–**I**) The prognostic values of ALDH6A1, ALDH3A1 and SLC7A8 in GC patients. OS: overall survival, FP: first-progression, PPS: post progression survival.

After picking out the three genes between the two datasets and the drug metabolism-associated gene dataset, we used the Kaplan-Meier plotter to investigate the prognostic values of ALDH6A1, ALDH3A1 and SLC7A8 in GC. Higher expression level of ALDH6A1 had a strong relationship with better OS (HR = 0.68, 95% CI = 0.58-0.81, p = 1.1e-05), FP (HR = 0.57, 95% CI = 0.47-0.7, p = 4.9e-08), PPS (HR = 0.57, 95% CI = 0.45-0.72, p = 2.4e-06) (Figure 2A–2C). In addition, high expression of ALDH3A1 was correlated with poor OS (HR = 1.2, 95% CI = 1.01-1.43, p = 0.035), FP (HR = 1.25, 95% CI = 1.02-1.53, p = 0.03), PPS (HR = 1.48, 95% CI = 1.19-1.85, p = 0.00045 (Figure 2D–2F). Meanwhile, as shown in Figure 2G–2I, high level of SLC7A8 was linked with poor OS (HR = 1.41, 95% CI = 1.17-1.69, p = 0.00021) and PPS (HR = 1.56, 95% CI = 1.22-2, p = 0.00042). Nonetheless, the expression level of SLC7A8 was not related to the FP (p > 0.05). Thus, we could suspect that ALDH6A1 had the great potential to be a prognostic prediction biomarker.

### The expression level of ALDH6A1 was downregulated in GC patients

**Figure 3:**
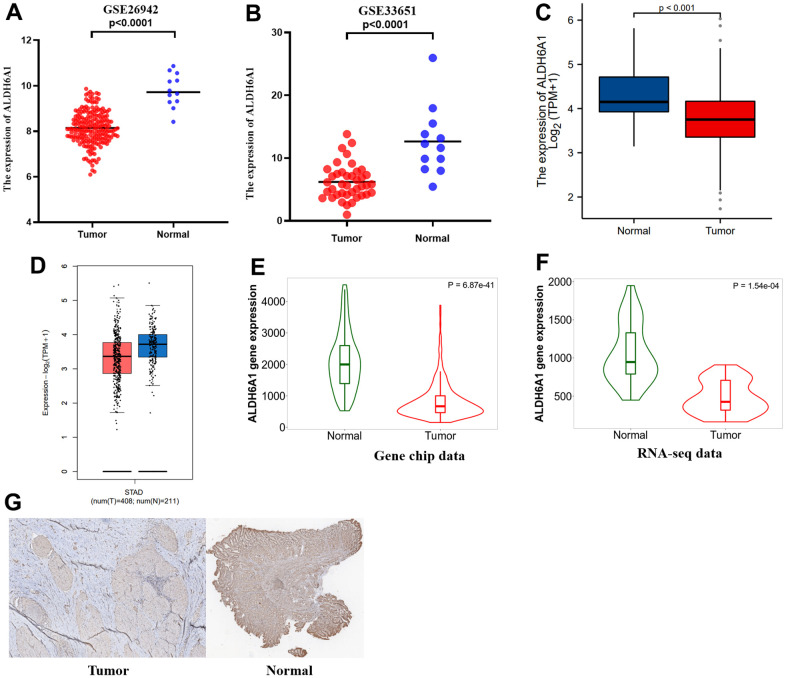
**ALDH6A1 expression was lower in GC group than in normal group.** (**A**, **B**) The expression levels of ALDH6A1 were lower in gastric cancer tissues when compared to normal gastric tissues. (**C**, **D**) Both the GEPIA2.0 platform and the TCGA platform showing ALDH6A1 expression was diminished in GC group. (**E**, **F**) The TNMplot platform has conveyed that ALDH6A1 expression decreased in GC group from RNA-seq data and gene chip data. (**G**) IHC analysis showed the ALDH6A1 expression was downregulated in GC tissues.

Two GSE datasets indicated that the level of ALDH6A1 expression was downregulated in gastric cancer (p < 0.0001) (Figure 3A, 3B). Likewise, the picture from Xiantao Xueshu (https://www.xiantao.love/) has conveyed that the expression of ALDH6A1 was lower in GC group when compared to normal group (Figure 3C). Meanwhile, the GEPIA2.0 database has demonstrated the same result (Figure 3D). Additionally, the TNMplot database has clarified that the gene expression level of ALDH6A1 in GC group was low both from the gene chip data (p = 6.87e-41) and RNA-seq data (p = 1.54e-04) (Figure 3E, 3F). Furthermore, IHC analysis was applied to further clarify the downregulated expression level of ALDH6A1 in GC tissues (Figure 3G). The above findings demonstrated the decreased levels of ALDH6A1 in GC. Then, we investigated the correlation between ALDH6A1 expression and the clinical characteristics of GC patients through TCGA database. We found that ALDH6A1 expression was significantly linked with T stage (p = 0.043) and antireflux treatment (p = 0.033) (Table 3).

### The co-expression network of ALDH6A1 in gastric cancer

**Figure 4:**
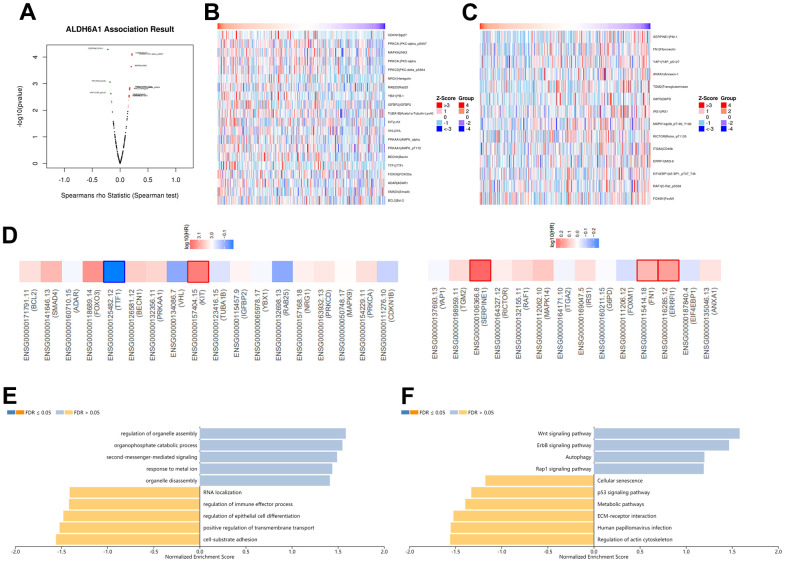
**The co-expression network of ALDH6A1 in gastric cancer.** (**A**) The LinkedOmics database has implicated the genes that possessed strong correlation with ALDH6A1 in GC. (**B**, **C**) The heatmaps has implied the top genes that owned positive and negative relationship with ALDH6A1 in GC. (**D**) The survival heatmaps has illustrated the top genes that were positively and negatively related to ALDH6A1 in GC. (**E**, **F**) GO and KEGG pathway of ALDH6A1 in GC.

In order to further explore the correlation between ALDH6A1 expression and the development of GC patients, the LinkedOmics database was employed. The plot has portrayed the co-expressed genes that were positively and negatively correlated with ALDH6A1 (Figure 4A). What’s more, there were 18 genes that had a positive link with ALDH6A1 (Figure 4B and Supplementary Table 2). Similarly, there were 14 genes that were negatively related to ALDH6A1 (Figure 4C and Supplementary Table 3). As shown in Figure 4D, the survival heatmaps illustrated the top genes that were positively and negatively related to ALDH6A1 in GC, including TTF-1, KIT, SERPINE1, FN1 and ERRFI1. Furthermore, after investigating the Gene Ontology signaling pathway, we could find that the co-expressed genes mainly took part in some biological processes, like regulation of organelle assembly, organophosphate catabolic process, cell-substrate adhesion and positive regulation of transmembrane transport, etc. (Figure 4E). Moreover, KEGG signaling pathway implicated that the co-expressed genes mainly took part in Wnt signaling pathway, ErbB signaling pathway, regulation of actin cytoskeleton and human papillomavirus infection, etc. (Figure 4F).

### The immune regulation of ALDH6A1 in gastric cancer

**Figure 5:**
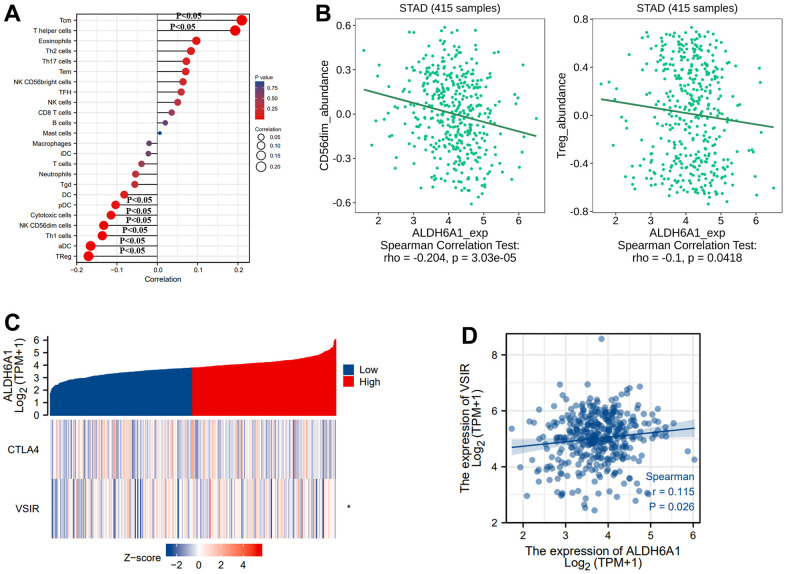
**The link between the ALDH6A1 expression and immune regulation of GC.** (**A**) The picture has conveyed the correlation between the expression level of ALDH6A1 and 24 types of immune cells. (**B**) The TISIDB platform portraying the link between ALDH6A1 and CD56dim cells, Treg (p < 0.05). (**C**, **D**) Both the heatmap and the scatterplot have illustrated that the ALDH6A1 expression was positively related to VSIR (p < 0.05).

In addition, we evaluated the correlation between ALDH6A1 expression and immune regulation of gastric cancer through the statistics obtained from the TCGA database. The diagraph has depicted that ALDH6A1 had a positive correlation with the infiltration of T helper cells and T central memory (Tcm). At the same time, ALDH6A1 was negatively linked with Treg, activated DC (aDC), Th1 cells, NK CD56dim cells, Cytotoxic cells, Plasmacytoid DC (pDC) (Figure 5A). Meanwhile, Figure 5B has portrayed that ALDH6A1 had a negative relationship with CD56dim cells (Spearman r = -0.204, p = 3.03e-05) and Treg (Spearman r = -0.1, p = 0.0418). Additionally, the pictures have illustrated that the expression level of ALDH6A1 was positively linked with VSIR (Spearman r = 0.115, p =0.026) (Figure 5C, 5D).

**Figure 6:**
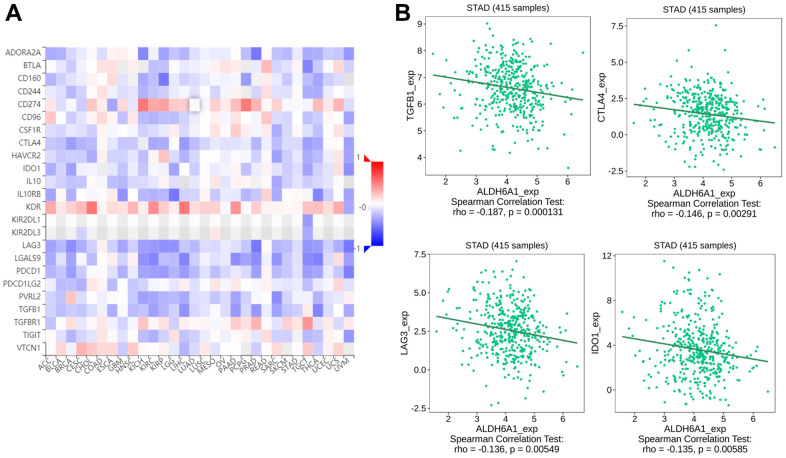
**The link between the ALDH6A1 expression and immunoinhibitors of GC patients.** (**A**) The picture has conveyed the relationship between ALDH6A1 expression and immunoinhibitors. (**B**) The scatter plots have depicted the top four immunoinhibitors which possessed a negative link with ALDH6A1 expression.

Similarly, we explored the correlation between ALDH6A1 expression and immunoinhibitors via the TISIDB database. The diagraph has illustrated the relationship between immunoinhibitors and the expression level of ALDH6A1 (Figure 6A). And the pictures have conveyed that the top four immunoinhibitors possessing the strong correlation with ALDH6A1 were TGFB1(Spearman r = -0.187, p = 0.000131), CTLA4 (Spearman r = -0.146, p = 0.00291), LAG3 (Spearman r = -0.136, p = 0.00549) and IDO1 (Spearman r = -0.135, p = 0.00585) (Figure 6B).

## DISCUSSION

In this paper, the link between the drug-metabolism related genes and the GC patients’ progression has been studied. Firstly, we downloaded two GC datasets from the GEO database and then explored the co-DEGs between the two GC datasets and the drug-metabolism associated gene dataset. And we figured out three downregulated genes through this way, including ALDH6A1, ALDH3A1 and SLC7A8. Likewise, the higher expression level of ALDH6A1 was related to the more favorable prognosis of GC patients. Meanwhile, the ALDH6A1 expression was higher in normal group than that in GC group. During the exploration of the co-expressed network with ALDH6A1, we figured out the genes owning the positive and negative correlation with ALDH6A1 in GC. The GO signaling pathway and KEGG signaling pathway have implicated that the co-expressed genes were related to the tumor microenvironment of cancer cells.

Drug metabolism has been demonstrated to be linked with the preclinical study of new drugs. The drug-metabolizing enzymes participated in the process of drug metabolism. To further investigate the drug metabolism in patients, scientists synergized the statistics and preclinical data *in vitro* and *in vivo* [21]. Interestingly, the deep study of drug metabolism, including drug sensitivity, could supply a novel insight into the targeted therapy of cancers [22]. ADH1B, a drug metabolism-related gene, was reported to play a vital part in the immune regulation of ovarian cancer patients [23]. Another study has demonstrated that a drug metabolism-related eight-gene signature, including ABCA1, ADH4, DHRS7, GPX3, NNMT, NOS3, SLCO4A1 and TAP1, was essential in the immune regulation of GC patients [24]. In GC tissues, there existed evidence that high mRNA level of the oxidoreductase ALDH6A1 could be relevant to the decrease of ROS and rise of NO. Furthermore, it could affect the cell viability environment and exert a double-edged sword effect on cancer cell apoptosis [25]. Intriguingly, another study has shown that ALDH6A1 expression was virtually higher in normal tissues than in cancerous tissues. Also, the ALDH6A1 expression decreased with cellular ageing, suggesting that this protein was also linked to cellular metabolism [26, 27]. The findings have conveyed that drug-metabolism associated genes played a significant part in GC patients’ progression. Exploring the relationship between GC prognosis and drug-metabolism associated genes could promote the development of GC therapy. And in this paper, we found that high expression of ALDH6A1 displayed favorable prognosis of GC patients.

ALDH6A1, as a potential target gene of HNF4A, could suppress the proliferation and metastasis of clear renal cell carcinoma (ccRCC). In addition, the expression levels of ABAT and ALDH6A1 were evidently diminished in ccRCC tissues [11, 28]. What’s more, ALDH6A1 and its isozymes were broadly correlated with cancers, implying that ALDH6A1 could be used for therapeutic targets [29]. Xu et al. have carried out a survey to inhibit the metastasis of breast cancer via CD44+/ALDH2+/ALDH6A1+ breast cancer stem cells (BCSCs) [30]. Additionally, through comprehensive evaluation of quantitative proteomic profiling and molecular features, a study found that ALDH6A1 expression decreased in hepatocellular carcinoma cells. The decrease of reactive oxygen species (ROS) and the elevation of nitric oxide (NO) in hepatocellular carcinoma were probably related to the low expression of ALDH6A1 [12]. Meanwhile, another study has shown that hypo-expression of ALDH6A1 was associated with survival of metastatic prostate cancer patients [31]. The downregulated ALDH6A1 has been proved to regulate the immune response in clear cell renal cell carcinoma, which implied a new biomarker for the clinical immunotherapy of ccRCC patients [32]. Our study is for the first time to figure out the drug-metabolism related gene ALDH6A1 expression was low in gastric cancer group.

A recent study has identified that anti-programmed death-ligand 1 and anti-programmed cell death protein 1 participated in the antitumor responses of GC patients at an advanced stage [33, 34]. Claudin 18.2 and the repairment of DNA damage could be new medicine target in gastric cancer. Triggering the immune response via PD-1/PD-L1 checkpoint inhibitors could provide new strategy in the treatment of gastric cancer patients [35]. Moreover, a study has implicated that besides the perioperative chemotherapy, immunotherapy also played an essential part in the third-line to the first-line of treatment of the gastric cancer patients [36, 37]. Our study has explored the relationship between ALDH6A1 and immune regulation of GC. ALDH6A1 was found to be negatively linked with immune cells, such as Treg, aDC, Th1 cells, NK CD56dim cells, cytotoxic cells and pDC. Simultaneously, ALDH6A1 was correlated with the immunoinhibitors, such as TGFB1, CTLA4, LAG3 and IDO1. The pDCs could cross-present antigens to CD8+ T cells and induce the response of melanoma-specific CD8+ T cells. Meanwhile, pDCs could suppress the antitumor immunity [38]. Through a study of 41 GC blood samples and 87 GC tissues samples, a study has reported that pDCs and ICOS+Foxp3+Treg cells could be prognostic prediction biomarkers of GC patients [39]. In this paper, we concluded that the expression level of ALDH6A1 had a positive correlation with VSIR. VSIR has been demonstrated to express in hematopoietic cells initially and VSIR could be a target of the cancer immunotherapy [40]. Furthermore, the Dies1/VSIR could trigger the differentiation of embryonic stem-cell and serve as an immune regulator through the BMP-pathway in gastric cancer [41]. These findings have illustrated that ALDH6A1 was related to the immune regulation of GC, which conveyed that ALDH6A1 could be a potential biomarker for future immunotherapy of GC.

## CONCLUSIONS

In summary, this article has demonstrated that ALDH6A1 expression was strongly correlated to the immune responses and prognosis of GC patients. What’s more, the expression level of ALDH6A1 was significantly linked with immunoinhibitory of GC. These findings have implicated that ALDH6A1, as a drug metabolism-associated gene, could be a new prognostic prediction strategy for the clinical treatment of GC patients.

## Supplementary Material

Supplementary Figure 1

Supplementary Table 1

Supplementary Tables 2 and 3
