# Supplementary material for: The downregulated drug-metabolism related ALDH6A1 serves as predictor for prognosis and therapeutic immune response in gastric cancer
Source: Aging (Albany NY). 2022 Sep 12;14(17):7038–51. doi: 10.18632/aging.204270 (PMC9512493; doi:10.18632/aging.204270)
Supplement: Supplementary Figure 1 [file aging-14-204270-s001.pdf]

SUPPLEMENTARY FIGURE

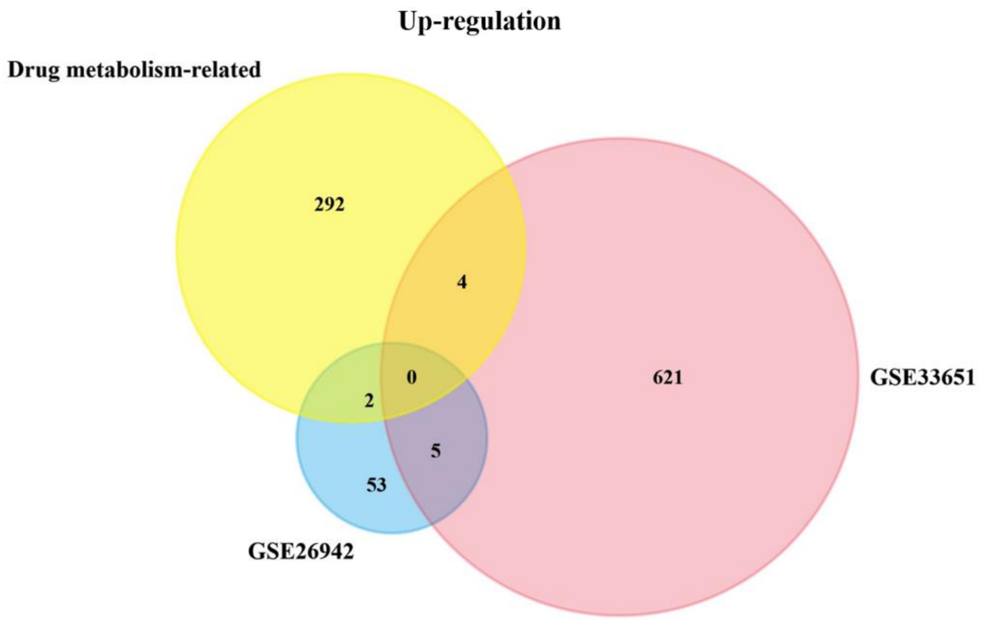

Supplementary Figure 1. Venn analysis portrayed the upregulated co-DEGs between drug-metabolism related gene dataset and two GC datasets. There existed no up-regulated co-DEGs between drug-metabolism related gene dataset and two GC datasets.
