# Supplementary material for: The downregulated drug-metabolism related ALDH6A1 serves as predictor for prognosis and therapeutic immune response in gastric cancer
Source: Aging (Albany NY). 2022 Sep 12;14(17):7038–51. doi: 10.18632/aging.204270 (PMC9512493; doi:10.18632/aging.204270)
Supplement: Supplementary Table 1 [file aging-14-204270-s002.docx]

**Supplementary Table 1. The upregulated and downregulated genes of the two datasets obtained from GEO database.**

**GSE26942，upregulated**

SHFM1

S100A3

CDA

ISG15

CEMIP

PKDCC

CLDN1

KRT17

FAP

CAMK2N1

LAMC2

TMEM158

BGN

RARRES1

TIMP1

ITGA5

IFI6

THY1

TUBB3

COL8A1

SPP1

PMEPA1

CDK11A

NKX3-2

CDH3

CST1

FNDC1

CLDN7

COL10A1

MSLN

HEPH

FOXC1

THBS2

SFRP4

SERPINE1

MMP11

TRIM15

TNFRSF12A

PRRX1

SERPINB5

CLDN3

SULF1

H19

KRT6B

PTGS2

COL11A1

THBS4

KLK6

CEACAM6

MMP3

IGF2BP3

EFNA2

PLA2G2A

CDX1

KRT7

HLA-DRB5

CLDN2

TFF3

REG4

COL1A1

**GSE26942，downregulated**

MMP24

DNHD1

AQP4

PTGDR2

ADH7

MFSD4A

ESRRG

SMIM11A

ALDH6A1

PDGFD

MRGPRD

CHRD

GLOD4

SLC2A12

TRIM50

CPA2

FGA

ETNPPL

NFE2L2

LIFR

SH3GL2

RAB11FIP2

ADHFE1

ATP4B

ADAM28

APOBEC2

KCNJ16

STX12

GCNT2

CLIC6

SLC9A4

KCNE2

CCDC121

DGKD

CKM

BTBD3

COLCA1

POSTN

ECI2

PRDM16

MYRIP

DERL3

IRF4

ATP11B

FAM189A2

GIF

XYLT2

TBC1D14

SLC26A9

SUCLG2

PIK3C2G

ATP4A

FUT9

GHRL

ATXN7L3B

NNT

KIT

FAR1

PP7080

PMM1

DRD5

INSIG1

CKMT2

PGA4

CCKBR

NTN4

NQO1

SIDT2

ALCAM

RNASE1

C18orf32

FBP2

SCNN1G

CHIA

14-Jul

SIGLEC11

PGA3

METTL7A

ARHGAP18

SNX24

GPER1

CHP1

LIPF

CYB5R1

DNER

CAPN13

ZNF385B

CMTM4

CKB

GSTA4

UBL3

RAP1GAP2

SPTSSB

SOSTDC1

PTGER3

PLPP3

COBLL1

PLCXD3

PTGR1

PSCA

ABCC5

CLCNKA

DHRS7

HDC

SLC9A2

CHPT1

TMEM171

POU2AF1

IRX3

C5

ARSD

PELI2

MYO10

TNFSF11

MYRF

AKAP1

GPRC5C

EPN3

LYPD6B

PDILT

SCNN1B

APLP1

ATP2A3

PAPSS1

FUT8

SULT2A1

CA9

ALDH3A1

CAMK1G

LRIG1

UQCRH

TRIM74

DUOX1

RBM47

RGMB

ARRDC4

CHGA

UGT2B17

HPN

CYFIP2

SOX21

SLC7A8

CYSTM1

RAB26

AMPD1

TNFRSF17

SLC4A2

GATA5

CDK12

TMED6

SORD

P2RX6

CAPN9

FBXL13

GPT2

TXNDC5

ARHGEF37

ZBTB7C

FA2H

TM7SF2

ALDH1A1

GCNT1

REP15

IRX2

UNC5CL

NPY

KLF4

GGT6

TSC22D3

PGA5

GSTA2

ADTRP

RASSF6

JCHAIN

IQGAP2

RNASE4

TMPRSS2

KCNJ13

AKR1B15

RPRM

ID1

LDHB

CXCL17

ADA

LDHD

SLC12A2

SORBS2

PXMP2

SH3BGRL2

ECHDC3

REC8

GC

VSIG1

SLC25A4

AKR7A3

TPD52L1

RAP1GAP

FAM3B

SLC22A23

HAS3

HABP2

MUC6

PTGS1

IGFBP2

COL9A2

STEAP3

KIAA1522

CHRM3

C1orf116

SGK1

FAM46C

GSTA1

DHCR24

ORM2

ITM2A

SELENBP1

ADH1C

SULT1C2

PLCXD1

CA2

RPS4Y2

VSIG2

KLK11

AKR1C2

ACSM3

CDH2

RNA28S5

GHR

SLC45A3

ME1

GKN2

ODAM

CA4

SLC39A11

BNIP3

VWA2

SOWAHA

SCGB2A1

ANKRD22

PLVAP

HPGD

CELA3A

CLDN18

FGG

MT1F

KIAA1324

CBS

KLRB1

CYP2C18

PROM2

MST1

TFF2

MECOM

CYP2S1

ADH1A

SOX2

TCEA3

MAOA

LEPR

MAL

AKR1B10

ST6GALNAC1

RAB27B

GKN1

CD36

TMEM97

COL4A5

PNPLA7

SSTR1

CXCL14

MT1E

AZGP1

FCGBP

CGNL1

MT1M

MT1H

AKR1C4

MZB1

SPDEF

ELOVL6

SDC4

PDIA2

HYAL1

MT1G

TMEM30B

FOXA1

CAPN8

GPX3

FMOD

ANG

REG3A

ARL14

VILL

ATP1B1

PLLP

DSC2

MAMDC2

CTSE

MRAP2

ABCA8

CD27

FAM3D

TCN1

KRT20

FOLR1

ANXA10

TFF1

FOXQ1

FMO5

SEPP1

TRNP1

PGC

AKR1C3

CYP4F12

TESC

C9orf152

FBP1

SERPINA5

HRASLS2

SMIM24

FABP4

STARD10

EIF1AY

SNORD13

BPIFB1

AADAC

SLC28A2

SST

SLC44A4

CYP3A5

HBB

CLDN23

SBSPON

PIGR

CD79A

GPT

SCNN1A

RPS4Y1

LTF

MLPH

AGR2

S100P

HBA2

FOXA3

PDK4

REG1A

SPINK1

NKX6-2

TSPAN1

HMGCS2

EEF1A2

PROM1

ALDOB

LCN2

DPCR1

PLA2G10

C6orf58

**GSE33651, upregulated**

KCMF1

RPL37

COL4A1

TCEAL4

MARS

VIM

IGFBP5

RGS1

ARID5B

RPL37A

TAOK3

CBWD3

KARS

ATRX

RPS24

PCMTD2

CTGF

NFYB

DHX58

UBC

PPIG

TALDO1

HLA-E

MS4A6A

NOMO3

RPL26

NCOA1

PHIP

NPM1

DNAJA1

SMARCA5

C4B

BCLAF1

CSNK2B

ZFP36L2

KLF10

LGALS1

ENG

PPP2CA

MDH2

RBPJ

RACK1

IARS2

LAMB1

AK6

MLKL

CALM1

C19orf53

COL1A2

CDV3

SLC15A3

EIF3F

FRG1

ETS2

TPM2

IVNS1ABP

CCDC71L

14-Jul

NRDC

CLTA

UTP14A

IPO5

RAP1A

LARP7

RSRP1

TARS

KRR1

GABARAP

BAZ2A

RPL5

TUBB6

PSMB7

ZNF394

KANSL2

DDX54

SRP14

C2

MRGPRX3

HSPA4

THY1

SENP6

GPX4

EIF3M

CAV2

COL3A1

RFX5

RGCC

TIE1

PLAU

ISCU

THEMIS2

ZNF7

ZHX1-C8orf76

FLII

TIMM9

TERF2IP

PSMD10

COL4A2

CCT8

COPRS

NARF

FTH1

PSMD7

GIMAP7

KCNJ8

RUBCN

IFI16

ADM

SIK1

RPL7

PELI1

LAMB2

USP39

OAZ1

NDUFA5

ADD2

DHX36

FN1

RPS27A

SLU7

NNMT

KIAA0040

SMARCA4

A2M

SNRNP35

FAS

POLR3C

IFT52

DUSP23

ARL5A

TOP1MT

RAB31

VPS16

PSMD2

CTSL

ENY2

RPS19

RSPRY1

TIMM50

PRPF40A

DDX5

FUNDC2P2

ATP6V1D

HTRA1

HTRA3

TNFSF10

FCER1G

ADAR

RBM38

C10orf128

TMSB4X

PRPF6

WDR53

UTP11

TUBA1C

GTPBP4

CTSK

AIDA

LGALS8

PPIAL4A

USP7

PTMA

AMZ2

UGP2

OLFML2A

ACSL4

LOC646014

NDUFAF5

GTF3C6

ADK

MAP4K5

RPUSD4

YBX1

CD248

FBXO32

DDX42

SH3BGRL3

EIF3A

CLCC1

POLR2B

CNOT10

MCM3AP

FNTA

IL15RA

ATG3

MFSD12

C19orf43

PLK2

EMILIN2

APOLD1

LOC105374313

GOLGA5

DDX49

IER5

LGALS12

UPF3B

CCDC91

NRBF2

SNUPN

TAF10

SLC43A3

COPS2

GPKOW

GNL2

SLC38A10

COL1A1

OXA1L

CHSY1

CCDC9

SON

RPL36

POP4

MGEA5

FAM120AOS

RBPMS

LOC100996724

STEAP4

TNFSF13B

DARS

PPAN

RAC2

FAM181A

FBLN5

SAP18

PAIP1

MEA1

UBR5

MS4A4A

RPL4

MAPK1

LAMA5

IFI30

IWS1

SERPINH1

WDR33

OCIAD1

PILRB

GLT8D1

CDKN2AIP

LPIN1

PLOD1

DLC1

CLPB

RAB34

GLRX3

UBA52

PSMB4

TIMP1

PTOV1

CPVL

ABRACL

TRIB1

ATPAF1

CCT3

SMG6

MRPL58

NELFE

UBE2R2

HUWE1

GBP1

SUMO2

PPM1M

CFLAR

ALDOAP2

RPL35A

AIMP2

SOD2

SERINC3

C1QB

C21orf33

GADD45B

SDHB

MAP4

SPTAN1

LSP1

GLIPR1

BAG3

DYSF

TAF3

CLEC2B

LOC100129550

RPL36AL

CD44

GDE1

KAT6A

DNAJC15

PHAX

LINC-PINT

PITPNA

BLMH

FAM222B

TYROBP

DNAJB12

LAPTM5

RBMS1

CD163

TRIM24

LOC100506990

TCEAL1

ANXA1

GYPC

SPARC

AKIP1

SNRK

PTPN2

TNIP1

CXCL10

AKTIP

MCM5

RRAGC

AKR1B1

COL6A2

HLA-DRB3

PIGS

TAF9

TMED5

RRAGA

TSN

SKAP2

NREP

NDUFB1

H3F3A

PDLIM4

PPIB

CHN1

CENPBD1P1

ZFP91

RAB9A

SDC2

DGCR6L

APMAP

RNF130

OLFM1

DECR1

HARS

SNX2

SH3BGRL

MGP

RBM17

COX14

TBCA

KIAA0922

GPAT3

TMEM2

TMEM176B

ZNF706

ITGAM

LRP11

GRASP

RPF1

HIST1H3C

MOB3A

POLR2K

RUVBL2

GIMAP6

RPAP3

KPNA4

COL15A1

CMSS1

RPL17

RAP1B

NOP56

LOC643733

IRF1

HIST3H3

COX6C

LMO2

NLRC5

USF2

SASH3

ESYT2

DUS1L

MRPL32

HCG18

FAM20C

PIPSL

VDAC3

AKAP12

LAT2

ATP6V1H

KIAA0196

ADGRA2

RPP25

VOPP1

NMI

MCUB

S100A9

ARHGDIB

MYO1B

ZFP36

DAP3

TCF3

CMTM3

GGCT

HLA-DPB1

CPSF1

TMEM204

FYB

NR4A1

STARD13

GNAI2

PLEKHG1

C1orf54

SUB1

SYNCRIP

BRK1

CETN3

SECTM1

NCAPD2

ATP5J2

ULK1

ANXA5

UBE2E3

PECAM1

TFB2M

GBP2

ANKRD10

CSF1R

S100A10

ACVRL1

IFITM2

CCDC85B

CAPNS1

ACTB

CTSB

SPAST

MYO9B

PPID

LOC105371433

ITGB2

PAPLN

ELOVL5

PQBP1

CLASRP

PAAF1

RGL1

POLE4

RGS2

ARHGEF17

PDIA3

EBNA1BP2

LOC105378591

SRGN

HSP90AB1

PROCR

RINL

SIKE1

SNRPG

CSRP1

ACTN1

PDLIM1

PMEPA1

ATPIF1

HOXB13

RDX

GAS5

MAP1LC3B

TRAF1

NDUFV2

CEBPB

PMP22

ATP1B3

RPL35

PPP1R12B

PKM

C21orf59

PSMC1

NAMPT

NCF4

IGSF6

CLNS1A

UBE2A

ERGIC3

GIMAP4

KLF6

PRKDC

EIF4EBP2

PTGDS

NR1H3

TMEM47

EXOSC8

CDK5RAP1

SLC25A33

PLEKHO2

SERPINA1

NAA20

IFITM4P

ALOX5AP

ANP32B

GPX7

IGSF3

CNPY2

SPSB2

EPHA2

ADAMTS5

PLEKHO1

BNIP3L

C1orf115

FLJ11710

CTSA

TMEM41A

ETS1

IFRD1

NATD1

TNS2

HEBP1

IFITM1

PCOLCE

FCGR2C

TIMM8A

C1R

RGS19

CCNB1IP1

RPL17-C18orf32

NCALD

INO80C

SPON2

NFKBIE

SMS

CLEC1A

HLA-DPA1

CFD

ST5

CCDC3

LAMP3

PER1

CNTNAP1

LAPTM4B

BTF3P9

GABARAPL1

PTGES3

SERBP1

DDX58

PMPCB

MAN1C1

MINOS1

HSPA1B

FTMT

MRPL24

FERMT2

TFG

BAG2

APOC1

TOMM70

S1PR3

WRNIP1

ATP6V1B2

LOX

BAD

ANKRD11

NOP58

PFDN2

EPSTI1

ASAH1

SH2D2A

SMTN

PSMD6

PLEK

TGFB1I1

ENPP2

SERPINA3

METAP2

ACAA2

HEG1

PARVG

LY6E

CFB

FTHL17

RNF146

RGS5

ADGRL4

MS4A7

DUSP6

NFKBIA

MYL9

CCDC80

C1QC

CENPB

DCAF13

TM4SF1

MAP1B

PPP6C

NAPRT

FAM102A

CASP4

CSF2RB

RTP3

RPS10P19

COX7A1

PRKCH

GLA

PSMD3

C1orf122

NDN

LTBP1

CHI3L1

UTP18

SPARCL1

RGMA

TNFSF15

FAM129A

RRS1

IL7R

ATP5A1

BST2

NEAT1

PLEKHF1

JAM3

IGF2

FOS

RARRES2

LOC100505942

CKS1B

UBD

PPP1R14A

SUPT20H

MEST

CRYAB

SLC6A8

SRPX

HOPX

DUSP2

CD55

CNN1

CDKN3

FABP4

CD68

ITGA7

IL1B

CAMK2N1

**GSE33651, downregulated**

RPL11

MIF

IKZF4

C9orf16

CD79A

UGCG

LINC01119

POU5F1P3

STARD4

DLX4

THADA

CYBA

RPS15

FOSL2

NCSTN

RIOK3

MRPL54

RBM3

GHSR

PPARG

SLC7A8

DPY19L2P3

HOOK2

MPDU1

IFI27

C14orf178

TAPBP

DGCR8

ACTG1

ARAF

NDUFC2

LOC101928291

PLXNB2

CERS6

SMIM14

SLC16A2

ATP2A3

TMEM14A

SELT

AHNAK

ALDOC

CPSF6

HPGD

HLCS

ALDH6A1

ACSS3

ARG2

IL17RE

SUCLG2

JUP

ARL6IP1

HES1

HNRNPL

RAB11FIP1

CLTB

SRSF10

F11R

RAB27A

TFF2

ALDH3A1

ANG

RNASE1

AKR1C2

MLPH

PROSER2-AS1

PBLD

C16orf89

TESC

LCP1

PGC

TFF1

DBP

FCGBP

In GSE26942, there were 60 up-regulated genes and 357 down-regulated genes. And, in GSE33651, there were 630 up-regulated genes and 73 down-regulated genes.
