# Supplementary material for: The downregulated drug-metabolism related ALDH6A1 serves as predictor for prognosis and therapeutic immune response in gastric cancer
Source: Aging (Albany NY). 2022 Sep 12;14(17):7038–51. doi: 10.18632/aging.204270 (PMC9512493; doi:10.18632/aging.204270)
Supplement: Supplementary Tables 2 and 3 [file aging-14-204270-s003.pdf]

SUPPLEMENTARY TABLES

Supplementary Table 2. The top 18 genes that are positively correlated with ALDH6A1 in gastric cancer.

CDKN1B	RAB25	VHL	ADAR
PRKCA	YBX1	PRKAA1	SMAD4
MAPK9	IGFBP2	BECN1	BCL2
PRKCD	TUBA1B	TTF1	
NRG1	KIT	FOXO3	

Supplementary Table 3. The top 14 genes that are negatively linked with ALDH6A1 in gastric cancer.

ANXA1	G6PD	RICTOR
EIF4EBP1	IRS1	SERPINE1
ERRFI1	ITGA2	TGM2
FN1	MAPK14	YAP1
FOXM1	RAF1	
